# Complete Mitogenomes of Xinjiang Hares and Their Selective Pressure Considerations

**DOI:** 10.3390/ijms252211925

**Published:** 2024-11-06

**Authors:** Ruijie Wang, Mayinur Tursun, Wenjuan Shan

**Affiliations:** Xinjiang Key Laboratory of Biological Resources and Genetic Engineering, College of Life Science and Technology, Xinjiang University, Urumqi 830046, China; wrj18832915559@163.com (R.W.); mayinuer0118@163.com (M.T.)

**Keywords:** mitochondrial DNA, phylogeny, hare

## Abstract

Comparative analysis based on the mitogenomes of hares in Xinjiang, China, is limited. In this study, the complete mitochondrial genomes of seven hare samples including four hare species and their hybrids from different environments were sequenced, assembled, and annotated. Subsequently, we performed base content and bias analysis, tRNA analysis, phylogenetic analysis, and amino acid sequence analysis of the annotated genes to understand their characteristics and phylogenetic relationship. Their mitogenomes are circular molecules (from 16,691 to 17,598 bp) containing 13 protein-coding genes, two rRNA genes, 22 tRNA genes, and a control region, which are similar with other *Lepus* spp. worldwide. The relative synonymous codon usage analysis revealed that the adaptation of *Lepus yarkandensis* to its unique arid and hot environment might be associated with synthesizing amino acids like alanine, leucine, serine, arginine, and isoleucine and the terminator caused by the different usage of codons. Further, we utilized the MEME model and identified two positive selection genes (*ND4*, *ND5*) in *Lepus tibetanus pamirensis* and one (*ND5*) in *L. yarkandensis* that might be important to their adaptation to the plateau and dry and hot basin environments, respectively. Meanwhile, *Lepus tolai lehmanni* and *Lepus timidus* may have evolved different adaptive mechanisms for the same cold environment. This study explored the evolutionary dynamics of Xinjiang hares’ mitochondrial genomes, providing significant support for future research into their adaptation mechanisms in extreme environments.

## 1. Introduction

The complex natural geography of Xinjiang Uyghur Autonomous Region (China) is characterized by a unique ‘three mountains sandwiching two basins’ topographical layout, with the Tianshan, Kunlun, and Altai mountain ranges encircling the major Tarim and Junggar inland basins [1]. This geography and the arid climate have shaped the complex and variable living conditions in this region [2]. Such an environment sets higher demands on the survival of the flora and fauna and provides a rich source of natural selective pressures for their evolution. Hares are one of the animals that have successfully adapted to such complex and diverse habitats. There are four hare species in Xinjiang: *Lepus yarkandensis* (Yarkand hare), *Lepus timidus* (Mountain hare), *Lepus tibetanus pamirensis* (Desert hare), and *Lepus tolai* (Tolai hare), with *L*. *tolai* comprising two subspecies, *Lepus tolai lehmanni* and *Lepus tolai centrasiaticus* [3,4]. *L. yarkandensis* and *L. tibetanus pamirensis*, as well as *L. yarkandensis* and *L. tolai*, are known to coexist in the junction of their distribution areas [4]. We also found individuals that exhibit morphological characteristics that are intermediate between two species [3]. *L*. *yarkandensis* and *L. timidus* are classified as national second-class protected animals of China, highlighting their significant role in the ecosystem [4,5], while *L. tibetanus pamirensis* and *L. tolai* have important economic value and contribute significantly to scientific research. Studying the adaptability and genetic evolution of these hares is crucial for understanding how they manage to survive and reproduce in such a harsh environment. The adaptability of *L. yarkandensis* to hot and arid environments has been analyzed by single nucleotide polymorphisms (SNPs) based on specific length amplified fragment sequencing (SLAF-seq), identifying genes that might be related to it [6].

The mitochondrial genome (mitogenome) evolution in mammals is faster than that of the nuclear genome [7]. Under evolutionary selection pressure, the mitochondria, lacking corresponding repair mechanisms, are prone to base mutations during the DNA replication process [8,9]. Mammalian mitochondrial genes are characterized by small genome size, stable gene content, relatively conservative gene arrangement, high information content, and maternal inheritance. These features have made them a powerful tool for studying animal molecular evolution, phylogenetic relationships, and conservation biology [10]. Mitochondrial genes are crucial to energy metabolism. Previous studies have indicated that extreme environmental conditions, such as low temperatures or oxygen levels, can drive adaptive changes in mitochondrial gene phosphorylation within populations [11,12]. These adaptive changes in response to the environment directly affect an individual’s survival and reproductive capacity. The selective pressure on mitochondrial genes in hares is highly pronounced in the alternating plateau–basin and hot–cold environments. By analyzing the selective pressure and changes at the genetic level, it is possible to reveal how species adapt to various environments through changes at the genetic level.

With the development of molecular biology techniques, some scholars began using mitogenome markers to explore the population genetics and taxonomy of *Lepus* species [13,14]. Previous studies based on the mitochondrial control region (CR) sequences of *L. yarkandensis*, *L. capensis*, and samples from unknown hare species found introgressive hybridization in the contact area between *L. yarkandensis* and *L. capensis* [15]. Shan [5] estimated the genetic diversity, genetic structure, and population history of the two Tolai hare subspecies, *L. t. lehmanni* and *L. t. centrasiaticus*, using SNP data from SLAF-seq of four mitogenome regions. Species-tree inference and coalescent simulations of 14 nuclear DNA and 2 mitogenome fragments from 11 hare species across Eurasia, North America, and Africa revealed recurrent mitogenome introgression in *Lepus* spp. [16]. The mitogenomes of many hare species have been successfully sequenced, including those of *L. yarkandensis*, *L. coreanus*, and *L. oiostolus* [14,17,18,19,20]. However, current mitogenome research on hares primarily focuses on the gene expression levels or individual mitogenomes, while comparative analysis of the mitogenomes among *Lepus* species is lacking. Studies on the environmental adaptability of Xinjiang hares using mitochondrial genomic data are also relatively lacking. Given the importance of mitochondrial genes to animal environmental adaptation, studying the entire mitochondrial genome of the *Lepus* species in Xinjiang is particularly important.

## 2. Results

### 2.1. Mitochondrial Genome Components and Sizes

**Figure 1:**
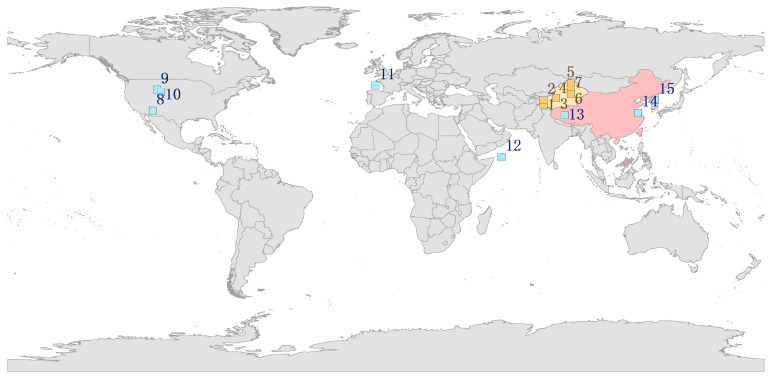
Global distribution of the *Lepus* species samples analyzed in this study. The red and yellow areas represent China, and the yellow area represents Xinjiang, China (GS (2024) 0650). Blue represents hares cited in GenBank, and yellow represents the Xinjiang hares. 1. *L. tibetanus pamirensis*, 2. Yarkand–Desert hare, 3. *L. yarkandensis*, 4. *Lepus timidus*, 5. *L. tolai lehmanni*, 6. *L. tolai centrasiaticus*, 7. Yarkand–Tolai hare, 8. *L. alleni*, 9. *L. americanus*, 10. *L. townsendii*, 11. *L. granatensis*, 12. *L. europaeus*, 13. *L. oiostolus*, 14. *L. capensis*, 15. *L. coreanus*.

**Figure 2:**
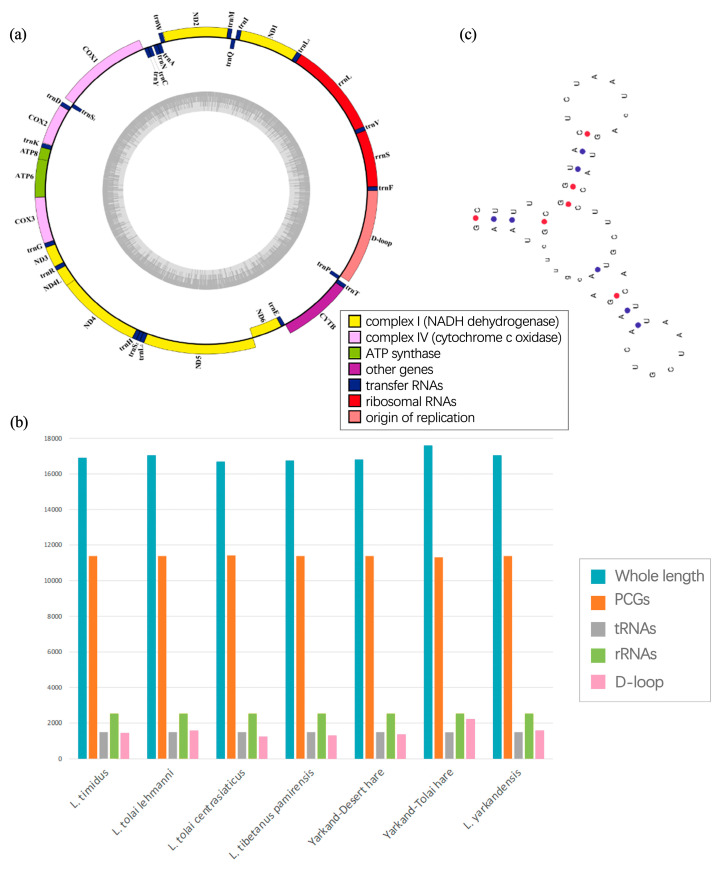
Mitochondrial genomes of Xinjiang hare species. (**a**) Circular structure maps of the mitochondrial genomes of 7 Xinjiang hares (16,691–17,598 bp). (**b**) Whole length and the lengths of protein-coding, tRNA, and rRNA genes, and the *D-loop* in the mitochondrial genomes of Xinjiang hares. (**c**) Secondary structure prediction diagram of *tRNA-Ser*.

The mitogenome sequences of the seven Xinjiang hares have been uploaded to GenBank: *L. timidus* (MN539745, 16,905 bp), *L. tolai centrasiaticus* (MN548102, 16,691 bp), *L. tolai lehmanni* (MN539744, 17,047 bp), the Yarkand–Tolai hare (MN539747, 16,809 bp), *L. yarkandensis* (MG279351, 17,047 bp), the Yarkand–Desert hare (LC073697, 17,598 bp), and *L. tibetanus pamirensis* (MN539746, 16,753 bp) (Figure 1 and Figure 2a). The average mitogenome length of the Xinjiang hares was 16,978 ± 305 bp (Supplementary Table S1). Among them, the mitogenome of the Yarkand–Desert hare was the longest, and that of *L. tolai centrasiaticus* was the shortest.

The mitogenomes of the *Lepus* spp. in Xinjiang are circular and show high similarity. They include 13 protein-coding genes (PCGs), 22 tRNA genes, and 2 rRNA genes, totaling 37 (Figure 2a and Supplementary Figure S1). The mitogenome also contains non-coding regions such as the CR (*D-loop* region) and intergenic regions. Most of the genes (28/37) are on the heavy strand (H strand), while *Nad6* and eight tRNA genes (*tRNA-Gln*, *tRNA-Ala*, *tRNA-Asn*, *tRNA-Cys*, *tRNA-Tyr*, *tRNA-Ser2*, *tRNA-Glu*, and *tRNA-Pro*) are on the light strand (L strand). Overlapping sequences and intergenic regions were found in multiple genes, with the largest gene overlap (43 bp) occurring between *Atp8* and *Atp6* and the largest intergenic region (average length of 32 bp) between *tRNA-Asn* and *tRNA-Cys* (Supplementary Table S2). We calculated the lengths of the PGCs, tRNA genes, rRNA genes, and CR and found that the main reason for mitogenome length differences was the CR length (Figure 2b).

### 2.2. tRNA and rRNA

The mitogenomes of the Xinjiang hares contain 22 tRNA genes, and all but *tRNA-Ser* can form the typical cloverleaf secondary structure of mammalian mitochondrial tRNA genes (Supplementary Figure S2). *tRNA-Ser*(AGN) lacks a recognizable dihydrouridine, forming a ‘two-leaf clover’ secondary structure (Figure 2c). In the mitogenome, the 12S rRNA (rrnS) is located between the *tRNA-Phe* and *tRNA-Val* genes, with an average length of 1578 ± 1.95 bp, and the 16S rRNA (rrnL) is between the *tRNA-Val* and *tRNA-Leu* genes, with an average length of 956 ± 0.49 bp. The rrnS and rrnL base lengths in the Xinjiang hares were very similar, differing by within 2 bp (Supplementary Table S3).

### 2.3. Base Composition and Skews

Analysis of the base content and skew in the mitogenomes of the 15 *Lepus* species found that the A + T content ranged between 60.5% (*L. americanus*) and 61.6% (*L. townsendii*). The AT skew values ranged between 0.028 (*L. europaeus*) and 0.042 (*L. oiostolus*). The GC skew values were all negative, ranging between −0.302 (*L. alleni*) and −0.328 (*L. townsendii*), reflecting the predominance of cytosine over guanine in *Lepus* species. It was evident from the base skews that A and C were more prevalent than T and G in the mitogenomes of all *Lepus* species assessed. The results showed significant similarity between *Lepus* species.

**Figure 3:**
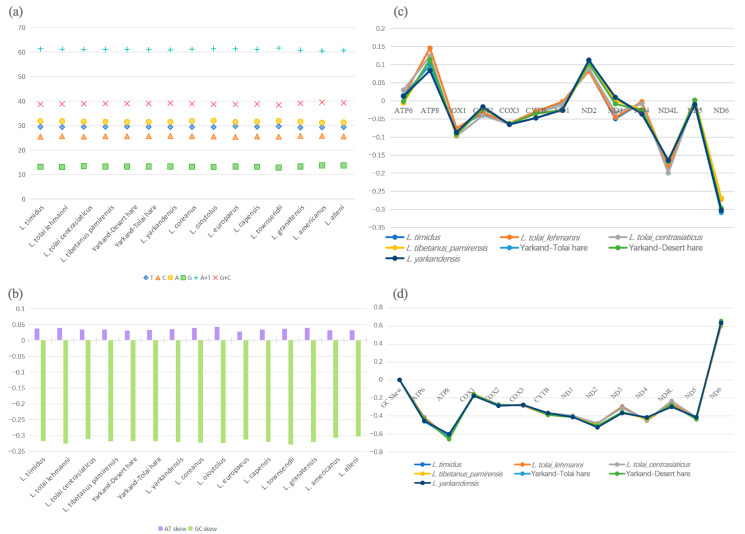
Mitochondrial genome base composition. (**a**) *Lepus* species mitochondrial genome base content. (**b**) *Lepus* species mitochondrial genome base bias. (**c**) Xinjiang hares’ protein-coding genes’ AT skew. (**d**) Xinjiang hares’ protein-coding genes’ GC skew.

The A + T content in the Xinjiang hares was mid-range within the *Lepus* genus, with *L. timidus* exhibiting the highest percentage (61.3%) and *L. yarkandensis* the lowest (60.9%). The A + T content for the Yarkand–Tolai hare hybrid fell between those of *L. yarkandensis* and *L. tolai centrasiaticus*. The Yarkand–Desert hare displayed a higher A + T content than both *L. yarkandensis* and *L. tibetanus pamirensis* (Figure 3a). *L. timidus* and *L. tolai lehmanni*, which inhabit the northern region, showed high AT skew values (0.038 and 0.039). The GC skew in the Xinjiang hares ranged between *L. tolai lehmanni* (−0.325) and *L. tolai centrasiaticus* (−0.302), the two subspecies of *L. tolai* (Figure 3b).

To further analyze the mitogenome composition of the Xinjiang hares, we calculated the AT and GC skew ratios of all the PCGs, tRNA genes, rRNA genes, and CR. All GC skew ratios were negative, and AT skew ratios were positive, except for the CR, in which the AT skew ratio was positive and the GC skew ratio negative (Figure 3c,d). All GC skew ratios for the PCGs were negative except for *Nad6*, which was positive. *Nad5* in *L. tolai lehmanni* and *Nad3* in *L. tibetanus pamirensis* had an AT skew ratio of 0, indicating equal amounts of A and T. The overall nucleotide composition of *Atp8* was significantly C-skewed in all *Lepus* species.

### 2.4. Relative Synonymous Codon Usage

**Figure 4:**
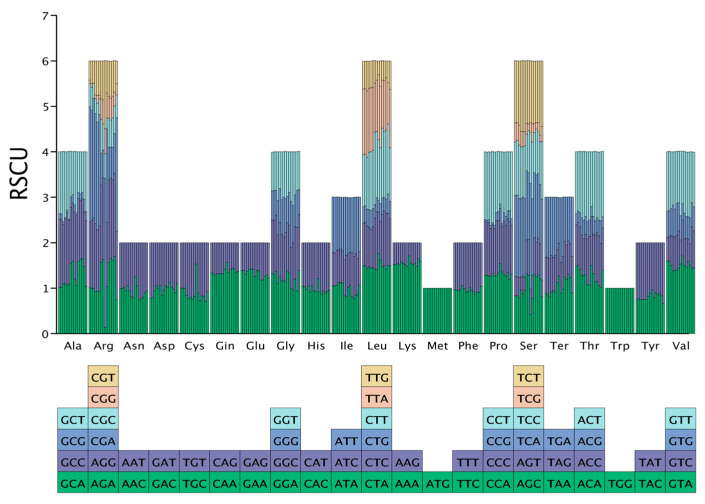
The relative synonymous codon usage (RSCU) in *Lepus* species worldwide. The columns from left to right show *Lepus timidus*, *L. tolai lehmanni*, *L. tolai centrasiaticus*, *L. tibetanus pamirensis*, Yarkand–Tolai hare, Yarkand–Desert hare, *L. yarkandensis*, *L. coreanus*, *L. oiostolus*, *L. europaeus*, *L. capensis*, *L. townsendii*, *L. granatensis*, *L. americanus*, and *L. alleni*.

We compared the relative synonymous codon usage (RSCU) values of various hare species to better understand the RSCU response to environmental changes during their evolutionary process. The RSCU exhibited remarkable consistency across species (Figure 4 and Supplementary Figure S3). However, *L. oiostolus* displayed distinct codon preferences that set it apart from its counterparts, particularly for the codons encoding arginine (Arg), cysteine (Cys), and serine (Ser). The differences in RSCU among the Xinjiang *Lepus* species were primarily evident in *L. yarkandensis*, which demonstrated a positive preference for utilizing the codons GCA (alanine, Ala), AGA (Arg), CTC (leucine, Leu), AGC (Ser), and TAA (terminator, Ter). These codons were subject to negative or no selection in the other Xinjiang hares. Conversely, the codons CGA (Arg), ATA (isoleucine, Ile), and TCC (Ser) were positively selected in the other Xinjiang hares but were negatively selected in *L. yarkandensis*. The selection of these special codons in *L. yarkandensis* resembled that in *L. capensis*. Furthermore, the CGG (Arg) codon was missing in *L. tolai centrasiaticus*.

Whether looking at the global hare populations or focusing solely on the internal diversity among the Xinjiang hare populations, the differences in RSCU were focused on the codons used for compiling Arg and Ser.

### 2.5. Phylogenetic Analysis

**Figure 5:**
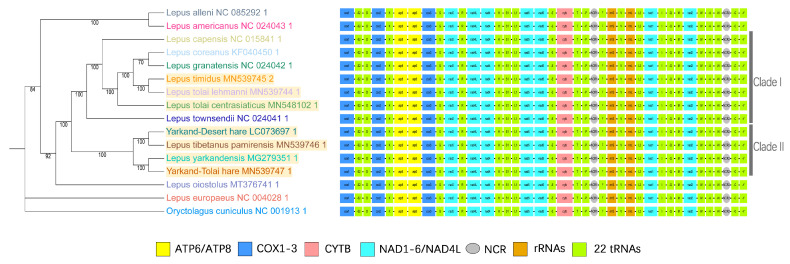
Phylogenetic tree, generated by IQ-Tree, and mitochondrial genome structure. The seven Xinjiang hares are shaded yellow. Each of the rectangular blocks represents a specific PCG in the mitochondrial genome, while the gray elliptical areas indicate non-coding regions.

A phylogenetic analysis of 16 mitochondrial genomes was conducted to investigate the evolutionary relationships of the Xinjiang hares (Figure 5). These contained 13 hare species, 2 hybrids, and *O. cuniculus* as an outgroup. This analysis revealed that the Xinjiang hares were clustered into two main branches. *L. tolai lehmanni*, *L. tolai centrasiaticus*, and *L. timidus* formed a single clade (Clade I), while *L. yarkandensis*, the Yarkand–Tolai hare, the Yarkand–Desert hare, and *L. tibetanus pamirensis* formed another clade (Clade II). Within Clade I, *L. tolai lehmanni* and *L. timidus* constituted a subclade with a closer relationship to *L. coreanus* and *L. granatensis* than to *L. tolai centrasiaticus*. Two subclades were identified in Clade II, one comprising *L. yarkandensis* and the Yarkand–Tolai hare and the other comprising the Yarkand–Desert hare and *L. tibetanus pamirensis*.

### 2.6. Control Region

The structural differences in the mitogenome of the Xinjiang hares were primarily observed in the non-coding regions, particularly the CR. Consequently, we further analyzed the CR, revealing a considerable variation in the full sequence length among species, ranging between 1248 and 2214 bp. We identified several highly conserved domains in the non-coding region of the investigated species, including the CSB I–III, the extended termination-associated sequence (ETAS), and the central CD (Supplementary Figure S4). The CD is a conserved region located downstream from the ETAS and contains several domains arranged in sequence. Characteristic motifs used to detect the CSB regions were GACATA for CSB I, CAAACCCCCC for CSB II, and TGCCAAACCCCAAAAAC for CSB III. The CD (317–318 bp) and ETAS (367–370 bp) regions exhibited relatively high levels of conservation, whereas the CSB regions (622–1371 bp) displayed a considerable degree of variation. We found repeated motifs in the CR of the Xinjiang hare’s mitogenome. A 22 bp consensus motif (CGTCTACGCGCACGTACACCCA) was repeated 4 times in the CR of *L. tolai lehmanni*, *L. tolai centrasiaticus*, and *L. tibetanus pamirensis* and 13 times in the CR of the Yarkand–Desert hare. Another 22 bp consensus motif (GCGCACGTACACCCACGTCTAC) was repeated 5 times in the CR of the Yarkand–Tolai hare and 13 times in the CR of *L. yarkandensis*. A third 22 bp consensus motif (GCGCACGTACACCCACGTTTAC) was repeated three times in the CR of the Yarkand–Tolai hare (Supplementary Table S4).

A comparative analysis of the composition of the ETAS, CD, and CSB regions in the CR of the Xinjiang hares’ mitogenome revealed that while C > T > A > G and G + C > A + T characterized the CD region, the other regions were characterized by A > C > T > G and A + T > G + C (Supplementary Table S2).

The CSB region in the Xinjiang hares comprised three main structural elements: the relatively conserved CSB I and CSB III, and the more variable CSB II. A short repeat sequence was found between CSB I and CSB II, and a long tandem repeat sequence followed the CSB III sequence. The pattern and copy number of the repeat sequences varied among hare species.

### 2.7. Selective Pressure on PCGs

**Figure 6:**
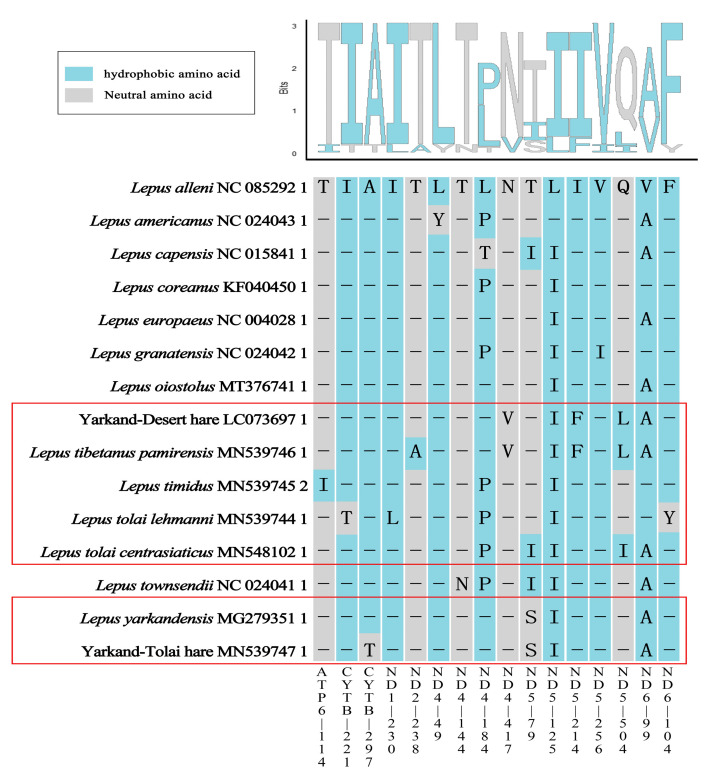
Shared selection pressure results in the protein-coding genes in *Lepus* species. The Xinjiang hares are enclosed in the red box. The bottom part shows the positively selected sites (*p* < 0.1). The gene location information of *L. alleni* was used as the reference sequence for the positively selected sites in the other species. The background color represents the amino acid properties. [Threonine (T); isoleucine (I); leucine (L); asparagine (N); valine (V); glutamine (Q); phenylalanine (F); tyrosine (Y); proline (P); alanine (A)].

We used MEME to analyze the selective pressure applied on individual codons in the PCGs of all 15 hare mitogenomes (Figure 6). Using the global hare data as a reference, we detected several sites under positive selection with the value of β+ (the rate of non-synonymous substitutions, dN) being greater than α (the rate of synonymous substitutions, dS) (*p*-value < 0.1; Supplementary Table S5). The Yarkand–Desert hare and *L. tibetanus pamirensis* exhibited the same uniqueness at multiple positively selected sites in the *ND4* and *ND5* genes (*ND4*-417: asparagine; *ND5*-214: isoleucine; *ND5*-504: glutamine), which differed from the other hares (*ND4*-417: valine; *ND5*-214: phenylalanine; *ND5*-504: leucine). Additionally, the positively selected *ND4*-184 (proline) in *L. timidus*, *L. tolai lehmanni*, and *L. tolai centrasiaticus* presented the same amino acid (leucine) in the other Xinjiang hares. The amino acid valine was detected at the positive selective site in *ND6*-99 in *L. timudus* and *L. tolai lehmanni*, differing from the alanine found in the other Xinjiang hares. The *ND5*-79 site in *L. yarkandensis* and the Yarkand–Tolai hare (serine) also differed from the threonine found in the other hares.

Selective pressure analysis on individual codons using only the Xinjiang hares as the background with the value of β+ being greater than α (*p*-value < 0.1; Supplementary Table S6 and Figure S5) found some selected sites that were also identified when the analysis was based on the global hare species, e.g., the *ND4*-417 site. We provisionally refer to these sites as commonly selected sites.

## 3. Discussion

### 3.1. Hare Mitochondrial Genome Phylogeny Analysis

The hare phylogeny often shows complex reticular relationships [21]. The Xinjiang hares in this study comprised two clades that were not grouped according to geographical distance. *L. tolai lehmanni* and *L. timidus*, collected from the same region, had the closest phylogenetic relationship but were more closely related to *L. granatensis* and *L. coreanus* than to the geographically closer *L. tolai centrasiaticus* (Figure 1). The Tianshan Mountains might be the geographical boundary between *L. tolai centrasiaticus* and *L. tolai lehmanni.* Our phylogenetic results are consistent with previous studies on the evolutionary relationships between *L. tolai* and *L. coreanus*, *L. timidus*, and *L. granatensis* based on mitochondrial and nuclear molecular gene fragments [16,21].

The similar appearance, introgression, and reticulated evolutionary relationships among hares have always made their classification a challenge [3,22]. In the past, when taxonomic techniques were not well developed, *L. tolai* and *L. tibetanus* in the Xinjiang region were mistakenly classified as *L. capensis* [23]. Evidently, it is difficult to accurately classify all hares based solely on morphology. In this study, two samples (the Yarkand–Desert and Yarkand–Tolai hares) appeared similar to both parent species and could not be classified accurately based on morphology alone. Phylogenetic analysis based on mitochondrial genomic data could help to estimate the taxonomic status of the two unidentified samples. At the mitochondrial genomic level, they were close to *L. tibetanus pamirensis* and *L. yarkandensis*, respectively (Figure 5). This finding supports referencing the mitochondrial genomic data in species classification, as shown in Modiolinae, in which morphological characteristics are inadequate for providing a definitive basis for classification [24].

### 3.2. Mitochondrial Genome Components

The results revealed that the gene organization in the mitogenome of the Xinjiang hares is the same as that of other *Lepus* species (Figure 5) and other mammals [14,25,26]. The AT skew, GC skew, and genomic nucleotide content revealed the base composition of their mitogenomes (Figure 3a,b). Consistent with the base composition of mitogenomes of other mammalian species, the AT content in *Lepus* was significantly higher than the GC content [27,28].

A comparison between the mitogenomes of Xinjiang and other *Lepus* species showed that the slight length difference was mainly due to differences in the CR. This finding is consistent with the fast evolutionary rate of the CR compared to protein-coding and rRNA regions, justifying its use to examine genetic variation in mammals [29]. The CR encompasses the CD, ETAS, and CSB regions (Supplementary Figure S4). A comparison of the CR in various mammals found that the ETAS1 sequence in most mammals (e.g., pig and antelope [30]) has a TCCCC motif [31]. This motif also appeared in the ETAS1 of *L. tolai lehmanni*, *L. tolai centrasiaticus*, and *L. timidus*. The other four Xinxiang hare samples had the ACCCC motif, as in horseshoe bats [32]. The motif sequence is GCCCC in some species in the Mustelidae, Bovidae, and Cervidae families [14,33]. We inferred that this sequence could be a recognition site to terminate *D-loop* synthesis [32]. The short tandem repetitive motifs observed in the CSB I and CSB II regions in *Lepus* spp. are considered a common feature in mitogenomes. We found that CSB I and CSB III were relatively conserved, while CSB II had considerable variations (Supplementary Table S3), unlike *Felis silvestris catus* and species in the Mustelidae family [31,33]. We suggest that the main factor affecting the length variation in the CR of the hare’s mitogenome is differences in the length of the CSB II region.

### 3.3. Adaptive Evolution

Codon usage bias is crucial to protein function and translation accuracy. Codon usage preference could affect the efficiency and accuracy of gene expression, thereby influencing protein function and the organism’s ability to respond to environmental changes [34]. We assessed the codon usage preference in the hare mitochondrial genome and found that, while most hares from Xinjiang had highly similar codon preferences, *L. yarkandensis* was unique in showing a preference for synthesizing Ala, Arg, Leu, Ser, Ter, and Ile (Supplementary Figure S3), similar to *L. capensis*. The *L. capensis* sample cited in this study came from Yancheng, Jiangsu Province, China, an area characterized by saline–alkali soil. Both *L. yarkandensis* and *L. capensis* live in arid environments, and both have a strong demand for regulating body water content. Ala is a nonpolar amino acid, and its high proportion in plants could help increase the thermal stability of proteins and reduce water loss, thereby helping them maintain their biological structure integrity under drought conditions [35]. Arg has multiple physiological functions in plants, including serving as a precursor to nitric oxide and polyamines, which are key to enhancing plants’ tolerance to environmental stress [36]. Leu is a hydrophobic amino acid whose expression could affect an animal’s drought resistance [37]. Ser contains a hydroxyl group and could be involved in forming hydrogen bonds that affect the three-dimensional structure and function of proteins [38]. Under drought conditions, Ser could help maintain the stability and function of proteins. Therefore, the particular codon usage bias in *L. yarkandensis* might be associated with its adaptation to arid habitats.

Regardless of whether the selective pressure analysis was conducted with or without the global hare species as a background, the *ND4* and *ND5* genes had many positively selected sites. *L. tibetanus pamirensis* (LC073697, MN539746) and *L. yarkandensis* (MN539747, MG279351) showed unique changes at multiple positively selected sites in these genes (Figure 6 and Supplementary Figure S5). Most positively selected sites were found in the *ND4* and *ND5* genes, resulting in mutations that produce hydrophobic amino acids [39]. ND4 and ND5 belong to the hydrophobic NADH complex I (ND1–6 and ND4L), one of the main complexes involved in the OXPHOS pathway in animals [40,41,42]. Previous studies have shown that positively selected sites are disproportionately concentrated in the NADH complex genes and affect the metabolic performance of organisms [40]. The mitochondrial OXPHOS pathway can simultaneously produce energy and heat; the balance between electron transport efficiency and heat generation benefits species’ survival in extreme temperatures [43].

Oxidative/reductive stress occurs in high-altitude areas where oxygen density is low. Studies on other mammals, such as camels living in the hot and arid Arabian region and animals adapted to other harsh environments, have shown that mitochondrial genes are under positive selection pressure [44,45,46]. In our study, *L. tolai lehmanni* and *L. timidus*, sampled from cold habitats, differed in the genes and sites under positive selection pressure. It is speculated that they use different molecular mechanisms for environmental adaptation. It is common for different species to achieve the same adaptive effect through different molecular mechanisms. For example, two positive selection signals in the *ND2* and *ND6* genes were found in the Sichuan golden snub-nosed monkey, but no such positive selection signals were detected in the Yunnan golden snub-nosed monkey, which is also a high-altitude species [47].

In this study, differences in positively selected sites in the PCGs of the Xinjiang hares may have resulted in differences in metabolic performances in different environments. The specific molecular mechanisms by which the Xinjiang hare mitochondrial genes under positive selection pressure support species adaptation to the environment need further research.

## 4. Materials and Methods

### 4.1. Sample Collection and Sequencing Assembly

This study used seven hare samples taken from various regions in Xinjiang between 2008 and 2019, including *L. timidus* (MN539745; 88° E, 47° N), *L. tolai lehmanni* (MN539744; 88° E, 47° N), *L. tolai centrasiaticus* (MN548102; 88° E, 43° N), *L. yarkandensis* (MG279351; 81° E, 40° N), *L. tibetanus pamirensis* (MN539746; 75° E, 37° N), and two samples whose morphological features were between two species and could not be clearly classified. These were recorded as the Yarkand–Desert hare (LC073697; 75° E, 39° N) and Yarkand–Tolai hare (MN539747; 88° E, 42° N) (Figure 1). Muscle or skin tissue samples were collected to extract the complete mitochondrial genomic DNA, whose integrity was determined using 1.0% agarose gel electrophoresis. The complete mitogenome was sequenced by next-generation sequencing at Hengchuang Gene Technology Co., Ltd., Shenzhen, China, using an Illumina Hiseq platform and assembled using SOAPdenovo2 [48].

The samples used in this study were provided to us by local forestry bureaus or came from hares that died of natural causes and from road accidents. Experimental protocols involved in this study were approved by the Institutional Animal Care and Use Committee of the College of Life Science and Technology, Xinjiang University, Urumqi, China (XJUAE-2023-020). The tissue samples were preserved in sterile tubes with anhydrous alcohol at −80 °C pending total mitochondrial genomic DNA extraction by a DNA tissue extraction kit.

### 4.2. Mitochondrial Genome Structure Analysis

We used the online tool OGDRAW (https://chlorobox.mpimp-golm.mpg.de/OGDraw.html (accessed on 2 September 2024)) to obtain the mitogenome structure diagram of the Xinjiang hare species [49]. The size of the mitogenome, CR, base composition of each component, gene arrangement order, gene overlaps, and intergenic regions of the seven Xinjiang hare mitogenome samples were analyzed using Mega11 [50]. The secondary tRNA structures were predicted and visualized using the tRNAscan-SE webserver [51,52].

### 4.3. Comparative Analysis of the Mitochondrial Genome of Lepus Species

Mitogenome sequences of the hare species were selected from GeneBank for comparative analysis. We excluded incomplete hare mitogenomes and prioritized those with ‘NC’, which were certified by NCBI, at the beginning. Eight *Lepus* mitochondrial genomes were obtained: *L. alleni*, NC 085292.1; *L. americanus*, NC 024043.1; *L. capensis*, NC 015841.1; *L. coreanus*, KF040450.1; *L. europaeus*, NC 004028.1; *L. granatensis*, NC 024042.1; *L. oiostolus*, MT376741.1; *L. townsendii*, NC024041 (Figure 1).

Combined with the sequencing data obtained from this experiment, we had mitochondrial sequence information of 15 hare species. A comparative analysis of genome size and structure was conducted. Mega11 was used to analyze the base composition of these mitogenomes and calculate the AT (A − T/A + T) and GC (G − C/G + C) skews to determine the bias in base content. The relative synonymous codon usage (RSCU) of the coding genes in the mitogenomes was calculated using the CAI module in Python [53]. A RSCU value = 1 indicated no preference for using that codon; RSCU < 1 indicated a lower degree of biased use of that codon; RSCU > 1 indicated a higher degree of biased use of that codon [54]. The results were visualized using R [55].

### 4.4. Phylogenetic Analysis

The 15 mitogenomes were subjected to phylogenetic analysis to determine the phylogenetic position of the Xinjiang hare species within the global hare population. This analysis included a rabbit mitogenome (*Oryctolagus cuniculus*, NC001913) from NCBI as an outgroup. First, the mitochondrial GeneBank data were extracted using Phylosuite v1.2.3 [56]. The most suitable model was selected using Partition Finder 2.1.1 [57]. Based on this model, the phylogenetic tree was constructed using IQ-Tree v2.3.6 [56] and visualized and annotated using iTOL v6 (https://itol.embl.de/login.cgi (accessed on 5 May 2024)).

### 4.5. Mitochondrial Genome Control Region

The CR is the main non-coding area in the mitogenome, containing the termination-associated sequence (TAS), a conserved domain (CD), and a conserved sequence block (CSB). Their structures were determined by comparing their sequences in the Xinjiang hares with those reported for other mammals. The start and end points of the CR were identified by locating the end of *tRNA-pro* and the start of *tRNA-phe*, respectively [14,58,59]. We found the CSB-F and CSB I of the Xinjiang hares by comparing the reported mitogenome CR sequences. Their start points were used as the boundaries for the TAS, CD, and CSB sequences. The base content, composition, and sequence structural characteristics of the CR and its parts were analyzed using Mega11.

### 4.6. Selective Pressure on PCGs

We used the web version of Hyphy’s DATAMONKEY (https://www.datamonkey.org (accessed on 24 September 2024)) for positive selection analysis of PCGs. The mixed effects model of evolution (MEME), which uses a mixed effects maximum likelihood approach to test the hypothesis of whether individual sites are under the influence of episodic positive or diversifying selection, was employed [60,61]. MEME is used to detect sites evolving under positive selection on a certain proportion of branches. For each site, MEME estimates two ω values and calculates the probability of evolving with each ω on a given branch. To infer ω, MEME infers α (dS) and two βs (dN), β− and β+. MEME enforces β− ≤ α in the null and alternative models. Therefore, β+ is the key difference between the null and alternative models. The null model requires that β+ ≤ α, while β+ is not restricted in the alternative model. When β+ > α, and the *p*-value < 0.1, the site is inferred to be under a significant positive selection. The likelihood ratio test was used to show selection significance, and the sites under positive selection pressure and variations in amino acids were analyzed.

## 5. Conclusions

We have successfully assembled seven complete mitogenomes of hares from Xinjiang, including four unique species, and performed extensive analysis to understand their characteristics and phylogenetic relationship. This study found that the mitogenome structure of *Lepus* species is highly conserved. The variations in mitogenome length are primarily attributed to differences in the CR length. Except for *L. yarkandensis*, the Xinjiang hares showed minimal differences in RSCU in their core PCGs. The phylogenetic results assisted us in determining the taxonomic status of samples that could not be accurately classified morphologically. We suggest that the *ND4* and *ND5* genes are associated with ecological adaptation in hares. However, different species appear to employ distinct adaptive mechanisms in similar environments. The positively selected sites at various protein-coding genes detected in the present study suggest that they may have evolved different metabolic adaptations in response to their contrasting environments. These findings will help to further investigate the *Lepus* mitogenome and will be valuable for future studies on hare adaptation to extreme environments in Xinjiang.

## Data Availability

The Mitochondrial Genome data presented in this study are openly available in the GenBank with the accession numbers MN539745, MN548102, MN539744, MN539747, MG279351, LC073697, and MN539746.

## Supplementary Materials

The following supporting information can be downloaded at https://www.mdpi.com/article/10.3390/ijms252211925/s1.
