# Supplementary material for: Complete Mitogenomes of Xinjiang Hares and Their Selective Pressure Considerations
Source: Int J Mol Sci. 2024 Nov 6;25(22):11925. doi: 10.3390/ijms252211925 (PMC11593953; doi:10.3390/ijms252211925)
Supplement: Supplementary file 1 [file ijms-25-11925-s001.zip › ijms-3270237-supplementary.pdf]

Supplementary Table S1. Mitochondrial gene sequence information.（The Xinjiang hares is marked in red.）

Accession number	Species	Length	T	C	A	G	A+T	G+C	AT skew	GC skew
NC004028	*Lepus_europaeus*	17734	29.8	25.4	31.5	13.3	61.3	38.7	0.027732463	-0.312661499
LC073697	Yarkand-Desert hare (*Lepus_tibetanus_pamirensis*)	17598	29.6	25.7	31.5	13.3	61.1	39	0.031096563	-0.317948718
NC085292	*Lepus_alleni*	16493	29.4	25.6	31.3	13.7	60.7	39.3	0.031301483	-0.302798982
NC024043	*Lepus_americanus*	17003	29.3	25.8	31.2	13.7	60.5	39.5	0.031404959	-0.306329114
MN539747	Lepus_yarkandensis_tolai (*Lepus_yarkandensis*)	16809	29.5	25.7	31.5	13.3	61	39	0.032786885	-0.317948718
NC015841	*Lepus_capensis*	17722	29.5	25.6	31.6	13.2	61.1	38.8	0.034369885	-0.319587629
MN539746	*Lepus_tibetanus_pamirensis*	16753	29.5	25.7	31.6	13.3	61.1	39	0.034369885	-0.317948718
MN548102	*Lepus_tolai_centrasiaticus*	16691	29.5	25.5	31.6	13.4	61.1	38.9	0.034369885	-0.311053985
MG279351	*Lepus_yarkandensis*	17047	29.4	25.8	31.5	13.3	60.9	39.1	0.034482759	-0.319693095
NC024041	*Lepus_townsendii*	17684	29.7	25.5	31.9	12.9	61.6	38.4	0.035714286	-0.328125
MN539745	*Lepus_timidus*	16905	29.5	25.5	31.8	13.2	61.3	38.7	0.037520392	-0.317829457
MN539744	*Lepus_tolai_lehmanni*	17047	29.4	25.7	31.8	13.1	61.2	38.8	0.039215686	-0.324742268
KF040450	*Lepus_coreanus*	17472	29.4	25.7	31.8	13.2	61.2	38.9	0.039215686	-0.321336761
NC024042	*Lepus_granatensis*	16915	29.2	25.8	31.6	13.3	60.8	39.1	0.039473684	-0.319693095
MT376741	*Lepus_oiostolus*	17370	29.4	25.6	32	13.1	61.4	38.7	0.042345277	-0.322997416
NC001913	*Oryctolagus_cuniculus*	17245	28.3	26.6	31.5	13.6	59.8	40.2	0.053511706	-0.323383085

Supplementary Table S2. Complete mitochondrial genome features of *Lepus* spp. in Xinjiang.

Gene name	strand	*L. timidus*			*L. tolai lehmanni*			*L. tolai centrasiaticus*			*L. tibetanus pamirensis*			Yarkand-Tolai hare			Yarkand-Desert hare			*L. yarkandensis*		
		MN539745			MN539744			MN548102			MN539746			MN539747			LC073697			MG279351		
		start	end	Intergenic	start	end	Intergenic	start	end	Intergenic	start	end	Intergenic	start	end	Intergenic	start	end	Intergenic	start	end	Intergenic
tRNA-Phe	H	1	68	-1	1	68	-1	1	68	-1	1	68	-1	1	68	-1	1	67	1	1	67	0
12S rRNA	H	68	1023	0	68	1023	0	68	1023	0	68	1023	0	68	1022	0	69	1024	0	68	1022	0
tRNA-Val	H	1024	1089	1	1024	1086	4	1024	1089	1	1024	1089	1	1023	1088	1	1025	1090	1	1023	1088	-2
16S rRNA	H	1091	2667	-1	1091	2667	-1	1091	2667	-1	1091	2670	-1	1090	2668	-1	1092	2671	0	1087	2668	0
tRNA-Leu (UUR)	H	2667	2742	2	2667	2744	0	2667	2744	0	2670	2747	0	2668	2745	0	2672	2746	2	2669	2743	2
ND1	H	2745	3699	-1	2745	3701	-3	2745	3699	-1	2748	3704	-3	2746	3704	-5	2749	3705	-2	2746	3702	-2
tRNA-Ile	H	3699	3769	-4	3699	3769	-4	3699	3769	-4	3702	3772	-4	3700	3770	-4	3704	3772	-3	3701	3769	-3
tRNA-Gln	L	3766	3837	9	3766	3837	9	3766	3837	9	3769	3840	9	3767	3838	9	3770	3841	9	3767	3838	9
tRNA-Met	H	3847	3915	0	3847	3915	0	3847	3915	0	3850	3918	0	3848	3916	0	3851	3919	0	3848	3916	0
ND2	H	3916	4959	5	3916	4959	5	3916	4959	5	3919	4962	5	3917	4962	3	3920	4963	5	3917	4960	5
tRNA-Trp	H	4965	5032	1	4965	5032	1	4965	5032	1	4968	5035	1	4966	5033	1	4969	5035	2	4966	5032	2
tRNA-Ala	L	5034	5100	0	5034	5100	0	5034	5100	0	5037	5103	0	5035	5101	0	5038	5104	0	5035	5101	-1
tRNA-Asn	L	5101	5173	31	5101	5173	33	5101	5173	31	5104	5176	33	5102	5174	34	5105	5177	32	5101	5174	32
tRNA-Cys	L	5205	5272	0	5207	5272	0	5205	5272	0	5210	5275	0	5209	5274	0	5210	5276	0	5207	5273	0
tRNA-Tyr	L	5273	5338	11	5273	5338	11	5273	5338	-8	5276	5341	7	5275	5340	7	5277	5342	7	5274	5339	7
COX1	H	5350	6891	2	5350	6891	2	5331	6887	2	5349	6890	2	5348	6889	2	5350	6891	2	5347	6888	2
tRNA-Ser(UCN)	L	6894	6962	3	6894	6962	3	6890	6958	3	6893	6961	3	6892	6960	3	6894	6962	3	6891	6959	3
tRNA-Asp	H	6966	7034	0	6966	7034	0	6962	7030	0	6965	7033	0	6964	7032	0	6966	7034	0	6963	7031	0
COX2	H	7035	7718	3	7035	7718	6	7031	7714	3	7034	7717	6	7033	7716	7	7035	7718	3	7032	7715	3

Continued Supplementary Table S2. Complete mitochondrial genome features of *Lepus* spp. in Xinjiang.

Gene name	strand	*L. timidus*			*L. tolai lehmanni*			*L. tolai centrasiaticus*			*L. tibetanus pamirensis*			Yarkand-Tolai hare			Yarkand-Desert hare			*L. yarkandensis*		
		MN539745			MN539744			MN548102			MN539746			MN539747			LC073697			MG279351		
		start	end	Intergenic	start	end	Intergenic	start	end	Intergenic	start	end	Intergenic	start	end	Intergenic	start	end	Intergenic	start	end	Intergenic
RNA-Lys	H	7722	7787	6	7725	7793	0	7718	7788	1	7724	7792	0	7724	7792	0	7722	7792	1	7719	7789	1
ATP8	H	7794	7997	-43	7794	7997	-43	7790	7993	-43	7793	7996	-43	7793	7996	-43	7794	7997	-43	7791	7994	-43
ATP6	H	7955	8635	-1	7955	8635	-1	7951	8631	-1	7954	8634	-1	7954	8634	-1	7955	8635	-1	7952	8632	-1
COX3	H	8635	9418	-1	8635	9418	-1	8631	9414	-1	8634	9417	-1	8634	9417	-1	8635	9419	-1	8632	9416	-1
tRNA-Gly	H	9418	9489	-1	9418	9489	-1	9414	9485	-1	9417	9488	-1	9417	9488	-1	9419	9488	0	9416	9485	0
ND3	H	9489	9835	0	9489	9835	0	9485	9831	0	9488	9834	0	9488	9834	0	9489	9835	0	9486	9832	0
tRNA-Arg	H	9836	9903	1	9836	9903	1	9832	9899	1	9835	9901	1	9835	9901	1	9836	9902	1	9833	9899	1
ND4L	H	9905	10201	-7	9905	10201	-7	9901	10197	-7	9903	10199	-7	9903	10199	-7	9904	10200	-7	9901	10197	-7
ND4	H	10195	11572	0	10195	11572	0	10191	11568	0	10193	11570	0	10193	11570	0	10194	11571	0	10191	11568	0
tRNA-His	H	11573	11641	0	11573	11641	0	11569	11637	0	11571	11639	0	11571	11639	0	11572	11640	0	11569	11637	0
tRNA-Ser (AGY)	H	11642	11700	0	11642	11700	0	11638	11696	0	11640	11698	0	11640	11698	0	11641	11699	0	11638	11696	0
tRNA-Leu (CUN)	H	11701	11770	-21	11701	11770	0	11697	11766	-21	11699	11768	0	11699	11768	0	11700	11769	0	11697	11766	0
ND5	H	11750	13582	-4	11771	13582	-4	11746	13578	-4	11769	13580	-4	11769	13580	-4	11770	13581	-4	11767	13578	-4
ND6	L	13579	14103	0	13579	14103	0	13575	14099	0	13577	14101	0	13577	14101	0	13578	14039	0	13575	14099	0
tRNA-Glu	L	14104	14171	3	14104	14171	3	14100	14167	3	14102	14169	3	14102	14169	3	14040	14107	3	14100	14167	3
CYTB	H	14175	15314	-1	14175	15314	-1	14171	15310	-1	14173	15312	-1	14173	15312	-1	14111	15256	-1	14171	15310	-1
tRNA-Thr	H	15314	15381	0	15314	15381	0	15310	15377	0	15312	15379	0	15312	15379	0	15256	15317	0	15310	15377	0
tRNA-Pro	L	15382	15447	0	15382	15447	0	15378	15443	0	15380	15445	0	15380	15445	0	15318	15383	0	15378	15443	0
D-Loop	H	15448	16905		15448	17047		15444	16691		15446	16753		15446	16809		15384	17598		15444	17047	

Supplementary Table S3. Nucleotide compositions and length for D-Loop in mitochondrial genome of *Lepus* spp. in Xinjiang.

Species	Accession number	Whole control region					Central domain					Conserved sequence blocks					Extended termination-associated sequences				
		Length (bp)	A%	T%	G%	C%	Length	A%	T%	G%	C%	length (bp)	A%	T%	G%	C%	Length (bp)	A%	T%	G%	C%
Lepus timidus	MN539745	1500	30.1	27.5	11.2	28.4	317	22	26.4	20.7	30	910	33.6	26.8	9.6	29.8	368	31.2	27.1	7.5	31.3
Lepus tolai lehmanni	MN539744	1600	31.2	27.9	11.3	29.7	318	22	27.4	20.4	30.2	912	33.8	27.7	9.5	28.9	370	32.7	28.6	7.6	31.1
Lepus tolai centrasiaticus	MN548102	1248	29.6	28	12.4	30	317	21.8	27.4	20.8	30	561	33.5	26.9	10	29.6	370	30.3	30	8.9	30.8
Lepus tibetanus pamirensis	MN539746	1308	29.7	27.8	12.4	30.2	317	22.4	27.1	20.5	30	622	32.3	25.6	10.5	31.7	369	31.4	32	8.7	27.9
Yarkand-Tolai hare	MN539747	1364	28.6	27.8	13.4	30.2	317	21.8	27.1	21.1	30	680	30.1	26.5	11.8	31.6	367	31.6	30.8	9.8	27.8
Yarkand-Desert hare	LC073697	2214	29.4	29.2	12.9	28.5	474	22.4	28.3	21.5	27.8	1371	31.4	28.8	11.1	28.7	369	31.4	31.7	8.7	28.2
Lepus yarkandensis	MG279351	1604	28.7	27.4	13	30.9	317	21.8	27.1	21.1	30	920	30	26.2	11.4	32.4	367	31.6	30.8	9.8	27.8

Supplementary Table S4. The structure and coordinates of *Lepus* spp. in Xinjiang.

Species	Accession number	Repeated motifs	Repeat times	ETAS	ETAS 1 (60bp)		ETAS 2 (63bp)		CDS	CSB	CSB 1 (25bp)		CSB 2 (17bp)		CSB 3 (18bp)	
					Coordinates	Structure	Coordinates	Structure			Coordinates	Structure	Coordinates	Structure	Coordinates	Structure
Lepus timidus	MN539745	ACACCCACGTACGCGCACGT	1	1-368	132	ACATACTATGTTTAATCGTGCATAAATTCCTCATCCCCATGAATAATAAGCTAGTACATTA	212	CACATACACCTGCCTAACTCCACAAAACCTTGTCATCAACACGAATATCTATATCCTACCACCCA	370-688	689-1601	731	TTTCTTTTAATGCTTGACGGACATA	960	AAACCCCCCTACCCC	1015	TGCCAAACCCCAAAAAC
Lepus tolai lehmanni	MN539744	ACACCCACGTACGCGCACGT/	4/	1-370	133	ACCATTCTATGTTTAATCGTACATTAAAACCTTATCCCCATGCATATAAGCTAGTACATCC	212	TACATTCCCTGCTAAACTCACAAACCCCTACCATCAACACGAATATCCATAACCCAATTACCCA	371-689	690-1602	730	TATCTTTTCATGCTTGACGGACATA	966	AAACCCCCCCTACCCCC	1041	TGCCAAACCCCAAAAAC
Lepus tolai centrasiaticus	MN548102	ACACCCACGTACGCGCACGT/	4/	1-370	133	ACCATTCTATGTTTAATCGTACATTAAAATCTCATCCCCATGCATATAAGCTAGTACATTC	212	CACATTCCCTGCTAAACCCACAAACTCCTATCCCCAACACGGATATCCTCAACCCTATCACCAA	371-688	689-1250	729	TATCTTTTCATGCTTGACGGACATA	895	AAACCCCCCCTACCCC	969	TGCCAAACCCCAAAAAC

Continued Supplementary Table S4. The structure and coordinates of *Lepus* spp. in Xinjiang.

Species	Accession number	Repeated motifs	Repeat times	ETAS	ETAS 1 (60bp) Coordinates	ETAS 1 (60bp) Structure	ETAS 2 (63bp) Coordinates	ETAS 2 (63bp) Structure	CDS	CSB	CSB 1 (25bp) Coordinates	CSB 1 (25bp) Structure	CSB 2 (17bp) Coordinates	CSB 2 (17bp) Structure	CSB 3 (18bp) Coordinates	CSB 3 (18bp) Structure
Lepus tibetanus pamirensis	MN539746	ACACCCACGTACGCGCACGT/	4/	1-369	132	ACCATTATATGTTTAATCATACATTAAAGCTTTACCCCATGCATATAAGCTAGTACATTC	210	CACATACACCTGCTTAACTCCACAAAACCTTGTCATCAACACGAATATCTATATCCTACCACCCA	370-687	688-1310	728	TATCTTTTCATGCTTGACGGACATA	954	AAACCCCCCCTACCCC	1028	TGCCAAACCCCAAAAAC
Yarkand-Tolai hare	MN539747	GCGCACGTACACCCACGTTTAC/GCGCACGTACACCCACGTCTAC	3/5	1-367	131	ACCATTATATGTTTAATCGTACATTAAAGCTTTACCCCATGCATATAAGCTAGTACATTC	209	CACATACACCTACTCAACTCCACAAAACCTTATCACCAACACGGATATTCAAACCCATTACCCA	368-685	686-1366	726	TATCTTTTCATGCTTGACGGACATA	1010	AAACCCCCCCTACCCC	1084	TGCCAAACCCCAAAAAC
Yarkand-Desert hare	LC073697	ACACCCACGTACGCGCACGT/	13/	1-369	132	ACCATTATATGTTTAATCATACATTAAAGTTTTACCCCATGCATATAAGCTAGCACATTC	210	CACATACACCTGCCTAACTCCACAAAACCTTGTCATCAACACGAATATCTATATCCTACCACCCA	370-844	845-2216	885	TATCTTTTCATGCTTGACGGACATA	1301	AAACCCCCCCTACCCC	1375	TGCCAAACCCCAAAAAC

Continued Supplementary Table S4. The structure and coordinates of *Lepus* spp. in Xinjiang.

Species	Accession number	Repeated motifs	Repeat times	ETAS	ETAS 1 (60bp) Coordinates	ETAS 1 (60bp) Structure	ETAS 2 (63bp) Coordinates	ETAS 2 (63bp) Structure	CDS	CSB	CSB 1 (25bp) Coordinates	CSB 1 (25bp) Structure	CSB 2 (17bp) Coordinates	CSB 2 (17bp) Structure	CSB 3 (18bp) Coordinates	CSB 3 (18bp) Structure
Lepus yarkandensis	MG279351	GCGCACGTACACCCACGTCTAC	13/	1-367	131	ACCATTATATGTTTAATCGTACATTAAAGCTTTACCCCATGCATATAAGCTAGTACATTC	209	CACATACACCTACTCAACTCCACAAAACCTTATCATCAACACGGATATCCAAACCCATTACCCA	368-685	686-1606	726	TATCTTTTCATGCTTGACGGACATA	1110	AAACCCCCCCTACCCCC	1183	TGCCAAACCCCAAAAAC

Supplementary Table S5. Selection pressure test results of shared protein-coding genes in Lepus species

Gene	Site	α	β-	β+	p-value
atp6	114	0	0	3651.05	0.06
cox2	133	0	0	599.76	0.09
cytb	221	19.32	0	5545.36	0.04
cytb	297	0	0	1150.6	0.07
nad1	230	0	0	17490.1	0.02
nad2	238	0	0	2218.73	0.06
nad4	49	22.11	0	3881.17	0.04
nad4	144	41.33	0	100000	0.03
nad4	184	0	0	67.92	0.07
nad4	417	33.81	0	1133.83	0.07
nad5	79	0.05	0.03	342.89	0.08
nad5	125	0	0	2330.63	0.09
nad5	214	0	0	2671.95	0.09
nad5	256	20.17	0	1186.55	0.08
nad5	504	0	0	2661.23	0
nad6	99	0	0	266.18	0.07
nad6	104	29.97	0	4006.17	0.06

Supplementary Table S6. Selection pressure test results of shared protein-coding genes in Xinjiang hares

gene	Site	α	β-	β+	p-value
atp6	59	0	0	1890.15	0.06
atp6	112	0	0	6122.25	0.05
cox1	332	0	0	1517.96	0.07
cytb	220	0	0	1817.62	0.06
cytb	296	0	0	937.54	0.09
nad1	228	0	0	12788.54	0.04
nad2	234	0.02	0.01	2301.35	0.08
nad4	414	0	0	1209.03	0.07
nad5	21	0	0	321.23	0.07
nad5	499	0	0	1777.01	0.07
nad6	104	64.27	0	4172.12	0.09

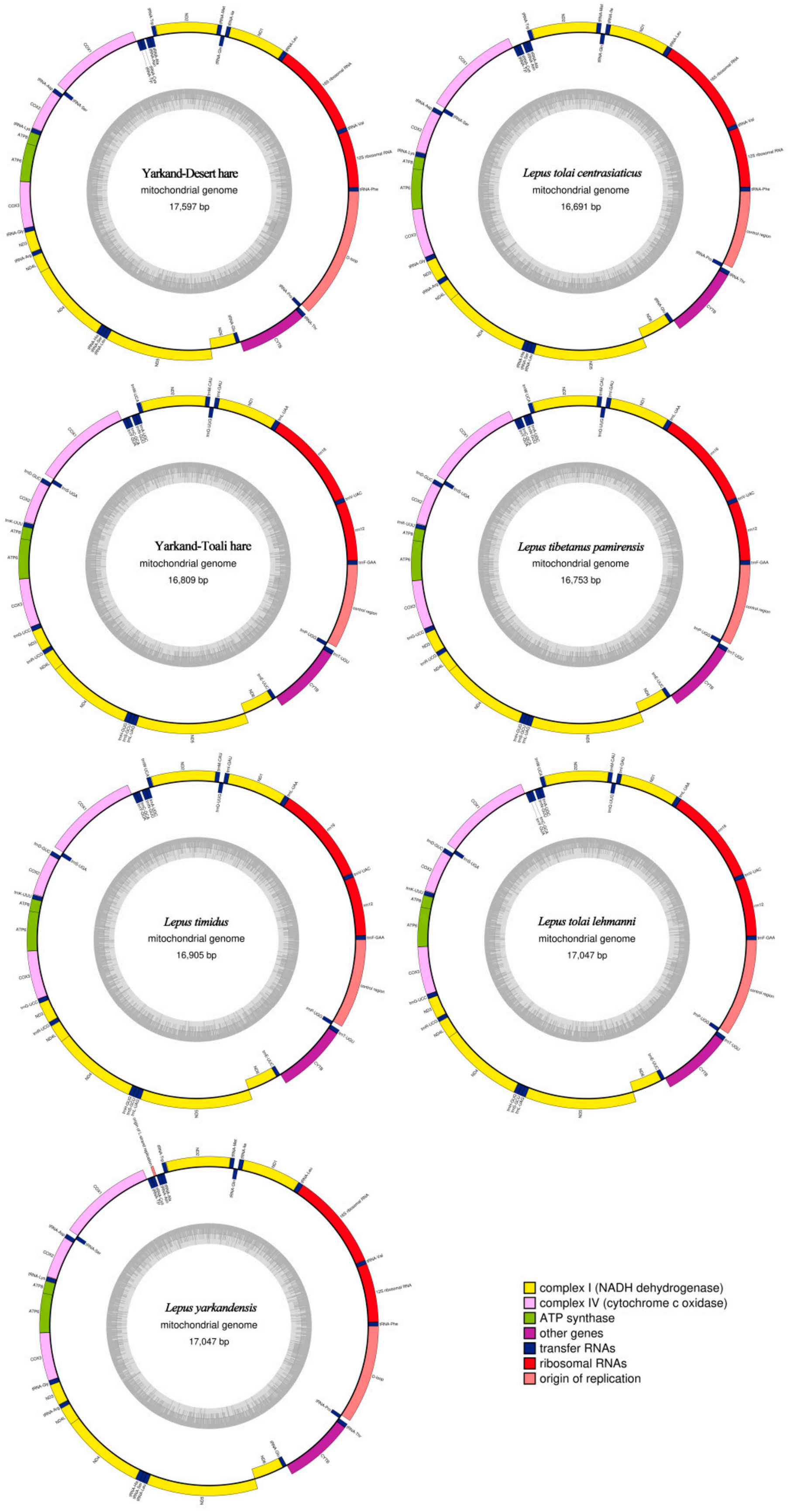

Supplementary Figure S1. Complete mitochondrial genome map of *Lepus spp.* in Xinjiang.

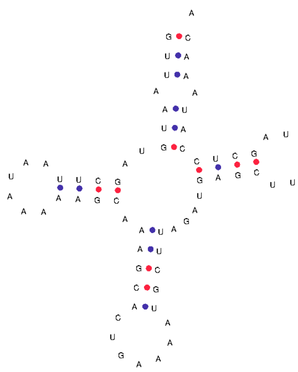

(1) tRNA-Phe

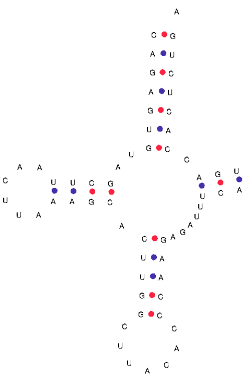

(2) tRNA-Val

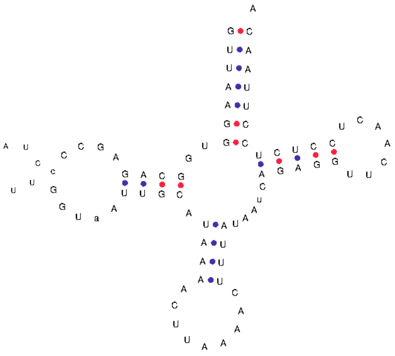

(3) tRNA-Leu

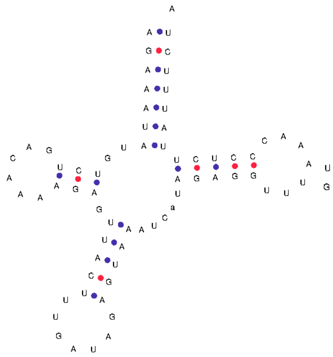

(4) tRNA-Ile

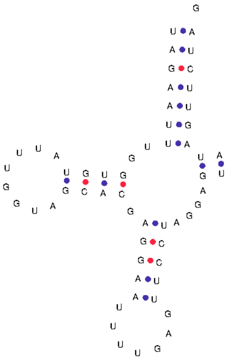

(5) tRNA-Gln

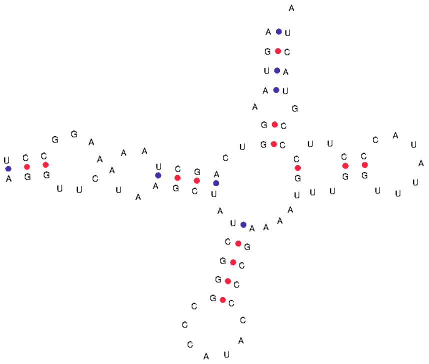

(6) tRNA-Met

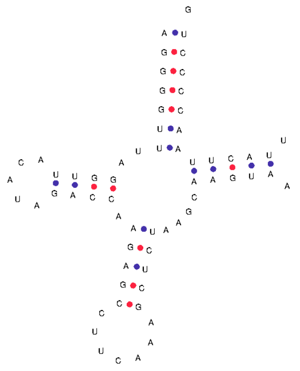

(7) tRNA-Trp

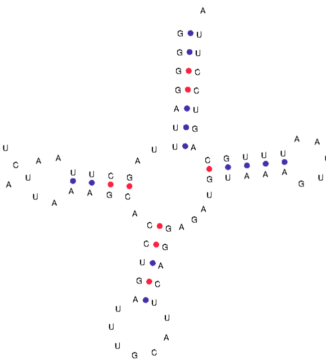

(8) tRNA-Ala

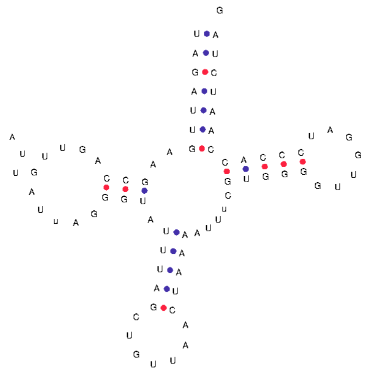

(9) tRNA-Asn

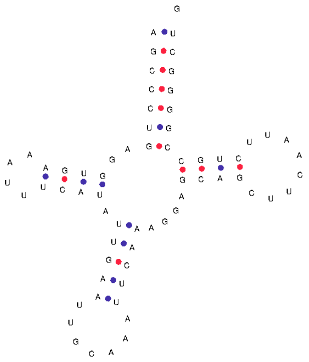

(10) tRNA-Cys

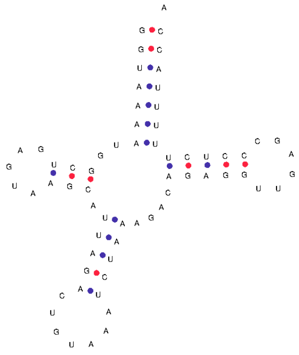

(11) tRNA-Tyr

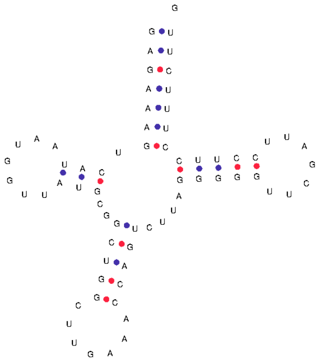

(12) tRNA-Ser

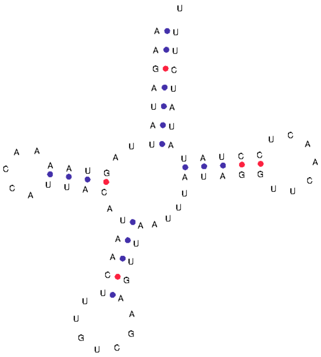

(13) tRNA-Asp

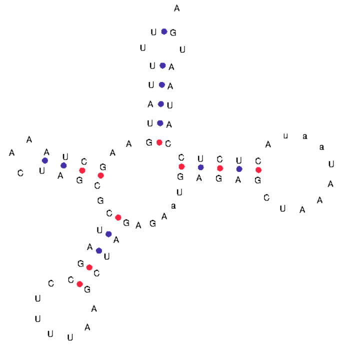

(14) tRNA-Lys

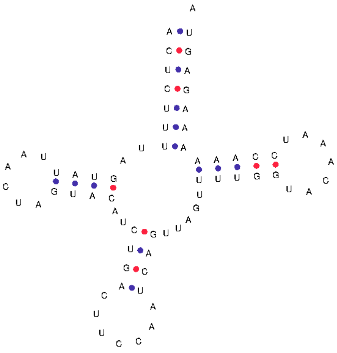

(15) tRNA-Gly

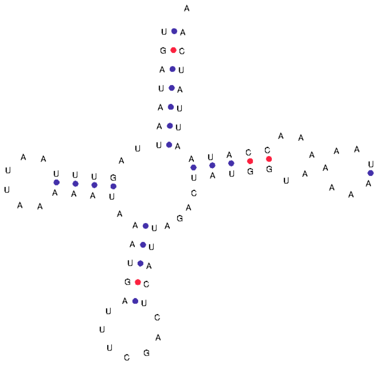

(16) tRNA-Arg

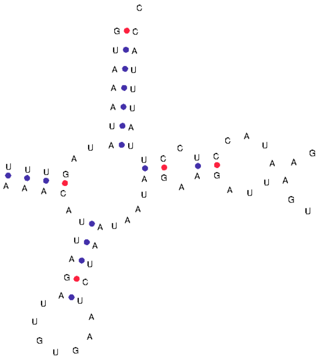

(17) tRNA-His

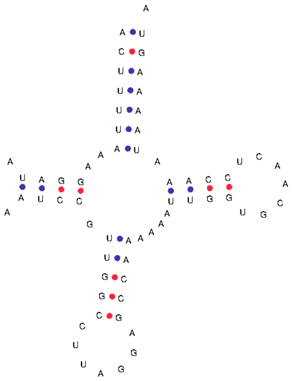

(18) tRNA-Leu

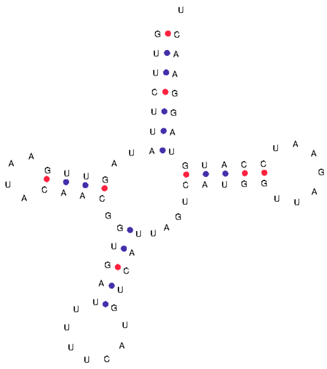

(19) tRNA-Glu

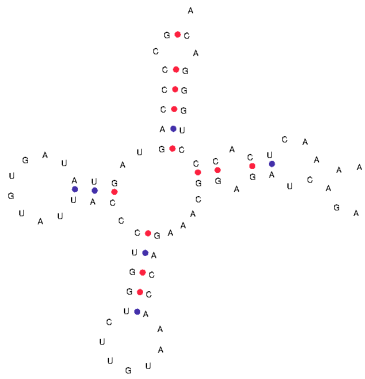

(20) tRNA-Thr

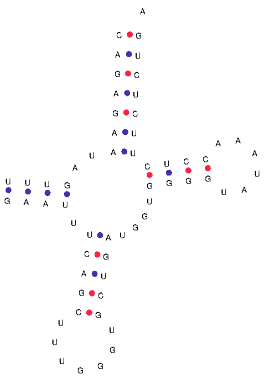

(21) tRNA-Pro

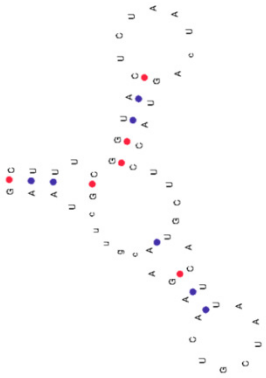

(22) tRNA-Ser

Supplementary Figure S2. The secondary structure of 22 tRNAs of the mitochondrial genome of *Lepus spp.* in Xinjiang.

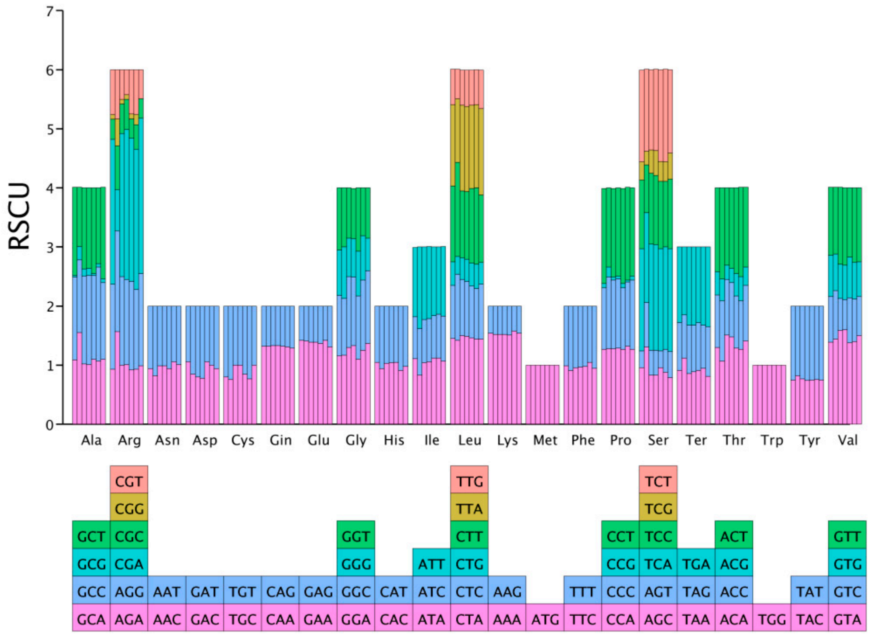

Supplementary Figure S3. Xinjiang hares RSCU Results. Each column from left to right is*:*Yarkand-Desert hare*, L. yarkandensis, Lepus tolai lehmanni, L. timidus, L. tibetanus pamirensis,* Yarkand-Tolai hare*, L. tolai centrasiaticus.*

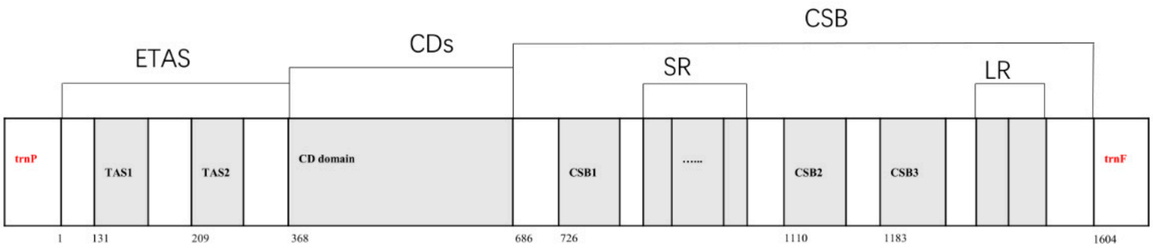

Supplementary Figure S4. Schematic diagram for control region in mitochondrial genome of *Lepus spp.* in Xinjiang.

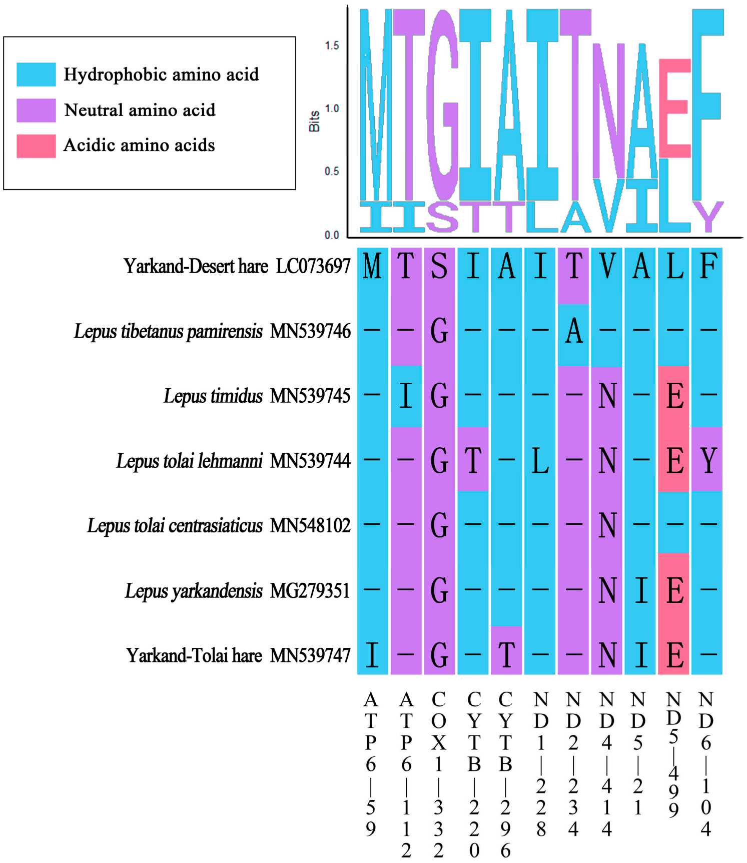

Supplementary Figure S5. Selection pressure test results of shared protein-coding genes in Xinjiang hares.The bottom part is the positively selected sites(P<0.1). All positively selected sites used the gene location information of Yarkand-Desert hare as the reference sequence.The background color represents the properties of amino acids.
